# Targeting Wild-type *NTRK* in *NTRK* Fusion–Negative Lung Cancer Decreases Brain Metastases

**DOI:** 10.1158/2767-9764.CRC-25-0811

**Published:** 2026-08-04

**Authors:** Maria J. Contreras-Zárate, Jenny A. Jaramillo-Gómez, R. Alejandro Marquez-Ortiz, Trinh C. Pham, Stella N. Koliavas, D. Ryan Ormond, Andre C. Navarro, Raphael A. Nemenoff, D. Ross Camidge, Diana M. Cittelly

**Affiliations:** 1 Department of Pathology, University of Colorado Anschutz, Aurora, Colorado.; 2 Department of Neurosurgery, University of Colorado Anschutz, Aurora, Colorado.; 3 Division of Renal Diseases and Hypertension, Department of Medicine, University of Colorado Anschutz, Aurora, Colorado.; 4 Division of Medical Oncology, Department of Medicine, University of Colorado Anschutz, Aurora, Colorado.

## Abstract

The central nervous system (CNS) is a common site of metastasis for both non–small cell and small cell lung cancer, yet the therapeutic strategies to prevent and decrease lung cancer brain metastases (BM) remain limited. Tyrosine kinase inhibitors have shown promising results in increasing overall response in BM, owing to their brain penetrance and increased effectiveness; however, their use is limited to a small group of tumors carrying specific oncogenic drivers. Among these, inhibitors targeting neurotrophic tyrosine receptor kinases (*NTRK*) are showing promising effects in reducing CNS metastases in cancers driven by gene rearrangements of these drugs’ targets. However, wild-type *NTRKs* are susceptible to activation by their canonical ligands, which are expressed throughout the BM niche and can, in a paracrine manner, activate *NTRK* function in cancer cells. In this study, we show that *NTRKs* are expressed in primary tumors, BM, and lung cancer cells with various driver mutations expressing wild-type *NTRK2* (WT-TrkB). We demonstrate that WT-TrkB activates downstream signaling and proliferation in response to exogenous brain-derived neurotrophic factor (BDNF) and conditioned media from reactive astrocytes known to secrete BDNF in the brain niche. Importantly, the FDA-approved *NTRK* inhibitor entrectinib blocked BDNF and astrocyte-induced survival pathways in multiple lung cancer cell lines, decreased their proliferation *in vitro*, and effectively prevented BM colonization and progression *in vivo* without significant effects on extracranial disease. Thus, these studies suggest that brain-dependent activation of *NTRK* is critical for BM of WT-*NTRK*^+^ lung cancers; therefore, *NTRK* inhibitors can be used to target nonfusion *NTRK* function to prevent or decrease BM.

**Significance:**

These studies demonstrate that *NTRK* wild-type receptors are important drivers of brain metastatic colonization and progression in different subtypes of lung cancer, independent of their driver alterations. Thus, they provide a rationale to expand the use of FDA-approved *NTRK* inhibitors with brain penetrance for the prevention of CNS metastases.

## Introduction

Brain metastases (BM) are a common site of metastatic spread for both non–small cell lung cancer (NSCLC) and small cell lung cancer, affecting 20% to 56% of patients with advanced lung cancer (1). The increase in lung cancer BM is likely the result of better neuroimaging capabilities and longer patient survival driven by effective new cancer treatments, which provide greater opportunities for the disease to spread to the brain. Treatment strategies for BMs, once dominated by local methods like surgery, whole-brain radiotherapy, and stereotactic radiosurgery, have been reshaped by the emergence of more effective systemic options, including targeted therapies, immune checkpoint inhibitors, and antibody–drug conjugates (2). However, the mortality associated with lung cancer BM progression remains high, and strategies to prevent or decrease central nervous system (CNS) involvement remain an urgent unmet need.

Among emerging therapeutic strategies for lung cancer BM, tyrosine kinase inhibitors have shown promising intracranial activity, in part due to their ability to penetrate the brain and effectively inhibit tumors driven by their specific oncogenic alterations (3). Importantly, inhibitors targeting the neurotrophic tyrosine receptor kinase (*NTRK*) family, which includes *NTRK1*, *NTRK2*, and *NTRK3*, encoding TrkA, TrkB, and TrkC, respectively, either as direct targets (e.g., Larotrectinib) or secondary targets (e.g., entrectinib, repotrectinib, lorlatinib, which also inhibit ALK and ROS1), offer new opportunities for tumors expressing wild-type *NTRK* receptors (4, 5). These receptors can be activated by their cognate ligands, which are highly abundant in the CNS (4, 6), supporting tumor growth within the brain microenvironment (7). However, the degree to which pharmacologic *NTRK* inhibition can affect lung cancer BM in tumors lacking *NTRK* gene rearrangements or other oncogenic targets of these drugs remains unclear.

Among *NTRKs*, wild-type TrkB (WT-TrkB) expression has been shown to promote lung adenocarcinoma metastases (8), and TrkB expression has been associated with worse survival in a panel of NSCLC samples containing squamous cell carcinomas, adenocarcinomas, and large-cell neuroendocrine carcinomas (9, 10). TrkB, in its native form, binds with high affinity to its cognate ligand brain-derived neurotrophic factor (BDNF) and, to a lesser extent, to neurotrophin-4. The protumorigenic function of BDNF/TrkB is well recognized in tumors with a propensity to colonize the CNS (breast, lung, and colorectal), playing critical roles in the ability of disseminated cancer cells to resist anoikis, survive, and invade at metastatic sites (8, 11–15). BDNF is expressed at a high level in certain regions of the brain, particularly the hippocampus, cerebral cortex, basal forebrain, and striatum, and its expression is strongly regulated by neuronal activity (16, 17). Astrocytes also secrete BDNF in response to multiple stimuli (18–21), and we have shown that breast cancer cells expressing wild-type TrkB activate downstream signaling in response to astrocytic BDNF and that the BDNF/TrkB inhibitor ANA-12 prevents brain metastatic colonization of TrkB^+^ breast cancer (BC) cells (7). Here, we demonstrate that TrkB and, to a lesser extent, TrkA are expressed in multiple lung cancer cells without *NTRK* rearrangements. WT-TrkB is activated in response to exogenous BDNF, astrocytic BDNF, and the brain niche and can be blocked to different extents with pan-TRK inhibitors. We show that astrocytes in the brain niche support the growth of lung cancer cells, and entrectinib, a pan-TRK inhibitor with high brain penetrance (22), can prevent and decrease the progression of non-*NTRK* rearranged lung cancer BM in preclinical models. Thus, these studies provide preclinical data to extend the use of *NTRK* inhibitors to target brain-specific survival pathways.

## Materials and Methods

### Cell lines

Human NSCLC cell lines LU65 (RRID: CVCL_1392) and H358 (RRID: CVCL_1559) were obtained from ATCC; both harbor heterozygous *KRAS*^G12C^ mutations. The murine Del19.1 cell line (*Egfr* Δexon19 deletion) was derived from a genetically engineered mouse model by our group (23). GFP^+^/luciferase^+^ derivatives were generated by transduction with the lentiviral vector pHAGE-EF1αL-luciferase-UBC-GFP-W, and GFP^+^ cells were selected by flow cytometry. These cell lines were cultured in RPMI-1640 supplemented with 10% FBS and 1% penicillin–streptomycin (P/S; complete medium).

The murine NSCLC cell line CMT167 (RRID: CVCL_2405), expressing the *Kras*^G12V^ mutation, was derived from mice (24). Its luciferase-positive (Luc^+^) derivatives were cultured in DMEM supplemented with 10% FBS, 1% P/S, and 20 mmol/L HEPES. Human brain-tropic NSCLC cell lines PC9Br3 (*EGFR* Δexon19 mutation) and H2030Br3 (*KRAS*^G12C^) were kindly provided by Dr. Sharon Pine and cultured as previously described (25). All human NSCLC cell lines were authenticated by the University of Colorado Cancer Center Cell Technologies Shared Resource using short tandem repeat profiling upon receipt. All experiments were performed using cells within 20 passages. Mycoplasma testing was performed every 3 months using MycoAlert PLUS (Lonza).

#### NTRK2 knockdown

Short hairpin RNAs (shRNA) targeting human TrkB (TRCN0000002242) and a nontargeting control (SHC002) were purchased from a Sigma Mission shRNA library. H2030Br3 cells were transduced and selected with puromycin. Knockdown efficiency was validated by quantitative RT-PCR. Total RNA was isolated using TRIzol, and cDNA was synthesized using the Verso cDNA Synthesis Kit (Thermo Fisher Scientific). RPLP0 was used as the normalization control. Relative mRNA expression levels were calculated using the comparative Ct (ΔΔCt) method. The primers used for validation were as follows: *NTRK2*: forward 5′-ACCCGAAACAAACTGACGAGT-3′ and reverse 5′-AGCATGTAAATGGATTGCCCA-3′ and RPLP0: forward 5′-GTGATGTGCAGCTGATCAAGACT-3′ and reverse 5′-GATGACCAGCCCAAAGGAGA-3′.

### Cell proliferation assays

NSCLC cells (2,000 per well) were seeded in 96-well plates in 100 μL of starvation medium and incubated overnight. The starvation medium was composed of phenol red–free DMEM (4.5 g/L glucose) supplemented with P/S, nonessential amino acids, sodium pyruvate, 0.1% BSA, and 5% charcoal-stripped FBS (cs-FBS). The next day, 50 μL of medium was removed and replaced with 50 μL of starvation medium containing either drugs (2×, 2 μmol/L) or vehicle (DMSO), resulting in a final concentration of 1 μmol/L. After 2 hours, treatments were applied with either BDNF (final concentration 50 ng/mL) or astrocyte-conditioned medium (Ast-CM; 20× stock diluted to a final concentration of 10×). Cells were imaged over time using the IncuCyte Live Cell Imaging system (Essen Bioscience). Cell confluence per well was quantified from four fields per well, with at least five replicates per treatment.

### Astrocyte cocultures and conditioned media

Primary neonatal astrocytes were isolated from P0–P2 CD1 mouse pups and seeded in T175 flasks and cultured as previously described (26). Astrocytes were cultured in regular medium (DMEM + 10% FBS + 1% P/S) until 100% confluence was reached, and microglia-free cultures were used for coculture experiments or obtaining conditioned media (Ast-CM). For *coculture experiments*, astrocytes were seeded in 12-well plates, grown to 100% confluence, and then cancer cells (10,000 cells/well) were seeded on top in 1 mL of starvation medium (DMEM supplemented with 2% cs-FBS) and incubated overnight. Treatments were applied at 2× concentration in 1 mL of serum-free starvation medium. Total green object area (μm^2^/Image) was quantified from 16 fields per well, with four replicates per treatment. To obtain conditioned media, T175 flasks at 100% confluence were rinsed with PBS and cultured in 25 mL of starvation medium for 72 hours. The conditioned media was collected, passed through a 0.2 μm filter, and concentrated 10× using 3 kD cutoff Amicon Ultra centrifugal filters (15 mL, UFC900324). Freshly prepared Ast-CM was used in all experiments.

### Human clinical samples (IHC)

Archival paraffin-embedded lung cancer BM samples were obtained from the University of Colorado Department of Pathology Biorepository under COMIRB protocol 15-1461. Formalin-fixed, paraffin-embedded sections were deparaffinized, subjected to heat-mediated antigen retrieval, and incubated with anti-TrkB antibody (Proteintech) overnight, followed by ImmPRESS anti-rabbit IgG secondary antibody (Vector Laboratories) for 30 minutes at room temperature. Detection was performed using DAB [DAKO kit; 1 drop (20 μL) per 1 mL substrate buffer]. Sections were counterstained with Vintage hematoxylin (1:1 dilution) for 1 minute, dehydrated, cleared, and mounted with Permount.

### Western blot

Cells were lysed at 4°C for 5 minutes in 2× RIPA buffer containing protease (Roche, #04693159002) and phosphatase inhibitors (Roche, #04906837001), followed by four 1-second sonication pulses at 20% amplitude. Protein concentration was measured using the Bio-Rad DC Protein Assay Kit II (#5000112). Between 20 and 40 μg of protein were resolved at 100 V on 8% to 10% SDS-PAGE or 4% to 15% precast gels (Bio-Rad, #4561086). Proteins were transferred to polyvinylidene difluoride membranes (Immobilon-FL 0.45 μm, Millipore, #IPFL00010 for >40 kDa; Hybond 0.2 μm, Amersham #10600022) and blocked with 3% BSA in TTBS for 1 hour at room temperature before overnight incubation with specific primary antibodies (Supplementary Table S1).

### Animal experiments

All procedures were approved by the University of Colorado IACUC (Protocol #00001227). Investigators were blinded to treatment groups. NOD/SCID gamma (NSG) mice (8–12 weeks old, male or female) received intracardiac injections of 200,000 H2030Br3 GFPLuc^+^ cells. *Preventive Study*: Ovariectomized female NSG mice supplemented with a 1 mg estradiol (E2) pellet were randomized to vehicle or entrectinib (ENT; 60 mg/kg, twice a day, orally) beginning 3 days before intracardiac injection and continued for 4 weeks. *Therapeutic Study*: NSG male and female mice were injected intracardially as above, and head bioluminescence signal was monitored every 10 days using an In Vivo Imaging System (IVIS). Mice with head signal ≥2× baseline 3 weeks after injection were randomized to receive vehicle or ENT (60 mg/kg, twice a day, orally) for 14 days. In all studies, metastatic burden was monitored weekly via IVIS.

#### *In vivo* luminescence (IVIS)

Mice received 200 μL of D-luciferin (GoldBio, LUCK-1G; 15 mg/mL) via s.c. injection. Head and extracranial metastatic burden were quantified using *Living Image* software (v2.60.1), utilizing the University of Colorado Anschutz Medical Campus Cancer Center Animal Imaging and Irradiation Shared Resource Facility (RRID:SCR_021980). At euthanasia, brains were incubated in 0.15 mg/mL luciferin in PBS for 10 minutes and imaged *ex vivo*.

### Histologic quantification

Micrometastases (<300 μm) and macrometastases (>300 μm) were quantified as described (27). Briefly, six hematoxylin and eosin–stained serial sections, 300 μm apart in a sagittal plane through one hemisphere, were analyzed at ×4 magnification using an ocular grid. Median counts per mouse were recorded.

### Immunofluorescence analysis

Fresh optimal cutting temperature–embedded hemisphere brains were sectioned at 20 μm thickness and stored at −80°C until use. Slides were thawed at room temperature and fixed in cold acetone for 5 minutes, followed by rinsing in Tris-buffered saline with 0.1% Tween 20 (TBST). Sections were blocked with 10% normal donkey serum for 30 minutes. Primary antibodies were incubated overnight (∼16 hours) under the conditions specified in Supplementary Table S1. Secondary antibodies were incubated for 1 hour at room temperature. Slides were mounted using Fluoromount-G containing DAPI (Invitrogen, 00-4959-52). Images were acquired using an Olympus APX100 with a 10× objective for whole-brain images and a 40× objective for regions used in analysis. Image quantification was performed using ImageJ.

#### Image analysis and quantification

Fluorescence images were analyzed using a custom macro in ImageJ, and the GFP channel was used to generate a segmentation mask. Thresholding of the GFP image was manually adjusted, after which the image was converted to a binary mask and refined using hole filling, dilation, and erosion. The resulting mask was converted to a region of interest and applied to the corresponding RFP image to measure mean RFP fluorescence intensity and area within the GFP-positive region. Measurements from all samples were automatically compiled into a single CSV file for statistical analysis.

### Publicly available data analysis

Gene expression data for lung adenocarcinoma (Project ID: TCGA-LUAD) were obtained from The Cancer Genome Atlas (TCGA) using the TCGA biolinks R package (28). Gene expression quantification counts were downloaded as normalized fragments per kilobase of transcript per million mapped reads. Expression profiles for *NTRK1*, *NTRK2*, and *NTRK3* were extracted specifically from samples categorized as tumor tissue.

The exported dataset was imported into GraphPad Prism version 10.6.1 for statistical analysis. Differences in transcriptional levels among the three *NTRK* genes were assessed using ordinary one-way analysis of variance (ANOVA) followed by Tukey multiple comparisons test to determine pairwise significance.

Spatial transcriptomic data were obtained from the publicly available GSE200563 dataset in the NCBI GEO (29). Raw digital spatial profiling counts were processed in R to correct for technical variability across areas of interest using 75th-percentile (Q3) normalization. Expression values for *NTRK1* and *NTRK2* were extracted and paired by patient identifier to enable direct comparison between primary tumors (PT) and their matched BM in patients with NSCLC (*n* = 23). Statistical analyses were performed using the Wilcoxon signed-rank test to evaluate paired differences in gene expression between PT and BM samples.

### Statistical analysis

Statistical tests and sample sizes are indicated in figure legends. Analyses were performed using GraphPad Prism v10.6.1. Data normality was assessed by Shapiro–Wilk and Kolmogorov–Smirnov tests. Parametric data were analyzed by one-way ANOVA or unpaired *t* tests; nonparametric data were analyzed by Kruskal–Wallis or Mann–Whitney U tests with appropriate multiple-comparison corrections. *P* ≤ 0.05 was considered significant.

## Results

### TrkB is expressed in lung cancer cells and is activated by exogenous BDNF

**Figure 1.**
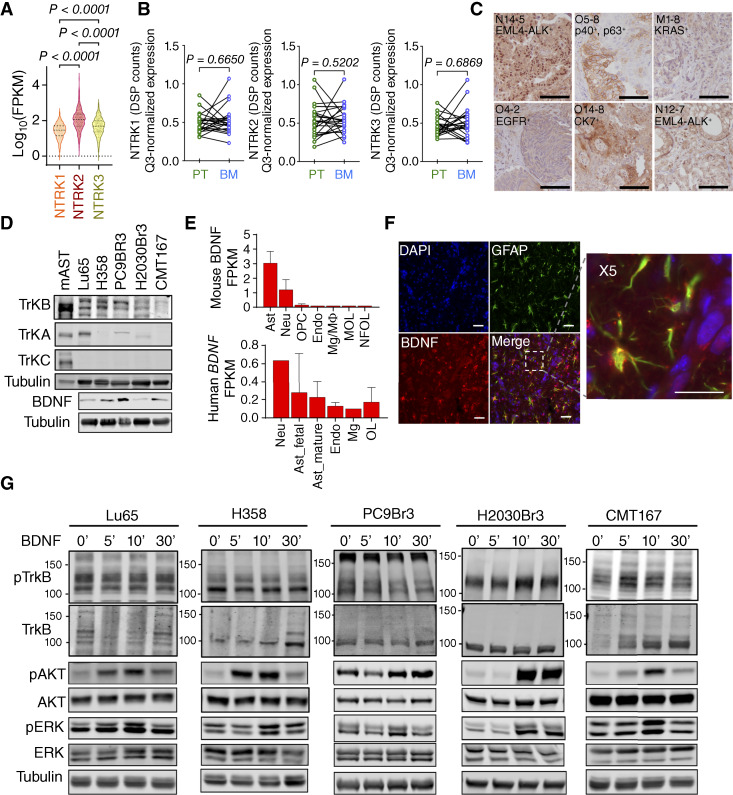
TrkB is expressed in lung cancer cells and activated by exogenous BDNF. **A,** Gene expression levels of *NTRK1*, *NTRK2*, and *NTRK3* in lung adenocarcinoma (LUAD) tumor samples from TCGA. Normalized gene counts [fragments per kilobase of transcript per million mapped reads (FPKM)] were obtained using the TCGA biolinks R package and analyzed in GraphPad Prism 10.6.1. One-way ANOVA with Tukey multiple comparisons. **B,***NTRK1*, *NTRK2*, and *NTRK3* expression between PTs and matched BM. Q3-normalized values were paired by patient ID (*n* = 23). Paired comparisons were performed using the Wilcoxon signed-rank test. **C,** IHC showing TrkB expression in a small cohort (*n* = 9) of NSCLC-BM; scale bars, 100 μm. **D,** Western blot showing expression of TrkB, TrkA, TrkC, and BDNF in NSCLC cell lines. Mouse astrocytes were used as a positive control for TrkA and TrkC. **E,** Relative mRNA expression of mouse and human BDNF in astrocytes (Ast), neurons (Neu), oligodendrocyte precursor cells (OPC), endothelial cells (Endo), microglia (Mg), macrophages (MΦ), oligodendrocytes (OL), mature oligodendrocytes (MOL), and newly formed oligodendrocytes (NFOL), data from Brain RNAseq.org. **F,** Immunofluorescence expression of BDNF in GFAP^+^ astrocytes surrounding lung cancer BM in a xenograft model. Scale bars, 50 μm. **G,** Western blots show the time course of BDNF-induced TrkB signaling in NSCLC cells serum-starved overnight and stimulated with 50 ng/mL BDNF. Tubulin served as a loading control in all blots. Molecular weight markers of 150 and 100 kD show full-length TrkB expression (110–140 kD depending on glycosylation levels).

We first sought to confirm the extent to which *NTRK* is expressed in patients with lung cancer and their BM. Analysis of publicly available datasets (TCGA–lung adenocarcinoma) showed *NTRK1*,* NTRK2*, and *NTRK3* mRNA expression in primary human lung adenocarcinoma, with higher expression of *NTRK2* mRNA (Fig. 1A). Comparison of *NTRK2* mRNA levels by spatial transcriptomic analysis (GSE200563) in a cohort of lung PTs and their matched BM (29) shows similar expression of *NTRK1*, *NTRK2*, and *NTRK3* in both primary and BM, suggesting *NTRK2* expression is a property intrinsic to the PTs(Fig. 1B). TrkB protein expression was confirmed by IHC in a small cohort of lung cancer BM (*n* = 9) without *NTRK* driver mutations (Fig. 1C). We confirmed that WT-TrkB is more prevalent than TrkA and TrkC across a panel of cell lines and detected variable expression of full-length TrkB (110–145 kD, consistent with differential glycosylation; ref. 30) in human (LU65, PC9Br3, H358, and H2030Br) and murine (CMT167) NSCLC cells, all of which lack *NTRK* rearrangements (Fig. 1D). As previously reported, BDNF is expressed in some lung cancer cell lines as well as in human and murine astrocytes in the normal brain (31, 32) and in reactive astrocytes surrounding lung cancer BM (Fig. 1E and F; ref. 7), suggesting that both autocrine and paracrine mechanisms may activate cancer cell *NTRK* signaling. To assess whether WT-TrkB is responsive to exogenous BDNF stimulation, we conducted a time course analysis of TrkB activation and downstream signaling in TrkB^+^ lung cancer cell lines. BDNF induced a robust activation of TrkB downstream signaling, characterized by increased phosphorylation of AKT and ERK within 5 to 10 minutes (Fig. 1G). Notably, TrkB and downstream AKT phosphorylation were observed in the absence of exogenous BDNF in some cell lines, especially PC9Br3, likely reflecting their high levels of endogenous BDNF expression (Fig. 1E). Moreover, shRNA–mediated downregulation of *NTRK2* in H2030Br cells (Supplementary Fig. S1A and S1B) reduced BDNF-induced downstream TrkB signaling, as evidenced by decreased phosphorylation of AKT and ERK (Supplementary Fig. S1C), further supporting a functional role for TrkB in these cells.

### ENT blocks BDNF-induced TrkB activation and proliferation of TrkB^+^ NSCLC cells without *NTRK* driving mutations

**Figure 2.**
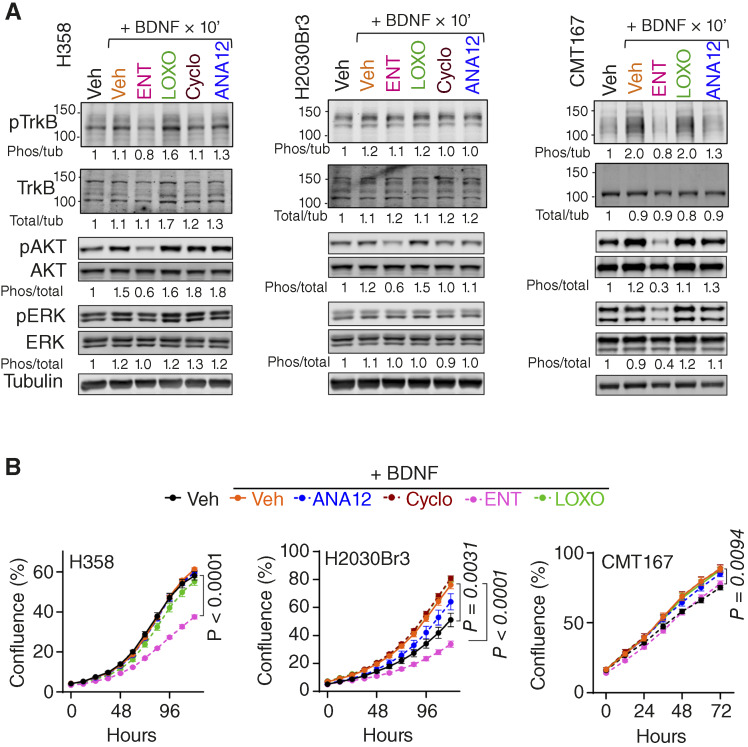
ENT blocks TrkB activation and proliferation of NSCLC TrkB^+^ cells lacking *NTRK* driving mutations. **A,** Western blot of NSCLC cells plated in phenol red–free media with 2.5% cs-FBS and pretreated for 2 hours with DMSO [vehicle (Veh)], ENT, LOXO101 (LOXO), cyclotraxin (Cyclo), or ANA12 (all drugs at 1 μmol/L), followed by stimulation with 50 ng/mL BDNF for 10 minutes. Tubulin served as a loading control. Densitometric values were calculated as phospho-TrkB/tubulin, total TrkB/tubulin, phospho-AKT/total AKT, and phospho-ERK/total ERK ratios and expressed relative to the Veh control group. **B,** Proliferation measured as percent confluency (mean ± SEM) using Incucyte live-cell imaging (*n* = 5 per treatment). Cells were plated in phenol red–free media with 2.5% cs-FBS and treated with Veh, 50 ng/mL BDNF alone, or BDNF combined with 1 μmol/L tyrosine kinase inhibitors. Data were analyzed by repeated-measures two-way ANOVA with *post hoc* multiple-comparison corrections.

To evaluate whether tyrosine kinase inhibitors targeting TrkB (ENT, LOXO 101, cyclotraxin, and ANA12) could block BDNF-induced TrkB signaling, H358, H2030Br3, and CMT167 cells were serum-starved overnight and pretreated with 1 μmol/L TKI for 2 hours prior to stimulation with exogenous BDNF for 10 minutes. Among the inhibitors tested, entrectinib effectively reduced BDNF-induced AKT and ERK activation in H358, H2030Br3, and CMT167 cells, but not in Lu65 or PC9Br3 cells (Fig. 2A; Supplementary Fig. S2A). In triple-negative breast cancer models (4T1Br5 and EO771), in which we had previously demonstrated that BDNF/TrkB signaling is important for proliferation and migration functions, entrectinib was also able to reduce BDNF-induced activation of AKT and ERK (Supplementary Fig. S2A), indicating the TRK-B blocking function of entrectinib encompasses multiple CNS-prone PTs.

To determine how BDNF/TrkB signaling affects the proliferation of lung cancer cells *in vitro*, cells were treated with BDNF alone or in combination with 1 μmol/L TKIs, and cell proliferation was measured via live cell imaging. Exogenous BDNF significantly increased proliferation in cells with low intrinsic BDNF expression, H2030Br3 and CMT167, but not in H358 cells. Consistent with the downstream inhibition of AKT in these cell lines, entrectinib significantly decreased their proliferation (Fig. 2B). By contrast, although entrectinib did not alter BDNF-induced AKT and ERK activation in LU65 and PC9Br3 cells at the time points tested (Supplementary Fig. S2B), sustained entrectinib treatment inhibited proliferation in these cell lines (Supplementary Fig. S2C). Notably, entrectinib decreased the proliferation of all cell lines (except for CMT167 cells) to levels below the vehicle control. Given that none of these cell lines express other entrectinib targets (ROS, ALK; Supplementary Fig. S2D), this suggests that entrectinib can target proliferation through BDNF-independent *NTRK* activity (Fig. 2B) and is consistent with its activity as an ATP-competitive kinase inhibitor able to shut down catalytic activity regardless of ligand availability, receptor conformation, or compensatory signaling feedback. ANA-12, a specific BDNF/TrkB competitive inhibitor is only effective in decreasing BDNF-induced proliferation in H2030Br cells, suggesting TrkB activation is an important driver of proliferation in these cells. Surprisingly, equivalent micromolar concentrations of other *NTRK* inhibitors (LOXO-101, cyclotraxin-B) were unable to effectively decrease signaling or proliferation in most cell lines, suggesting entrectinib achieved robust pathway inhibition at the same dose.

### ENT blocks proliferative signaling and cell proliferation of NSCLC cells in models mimicking interactions with the brain TME

**Figure 3.**
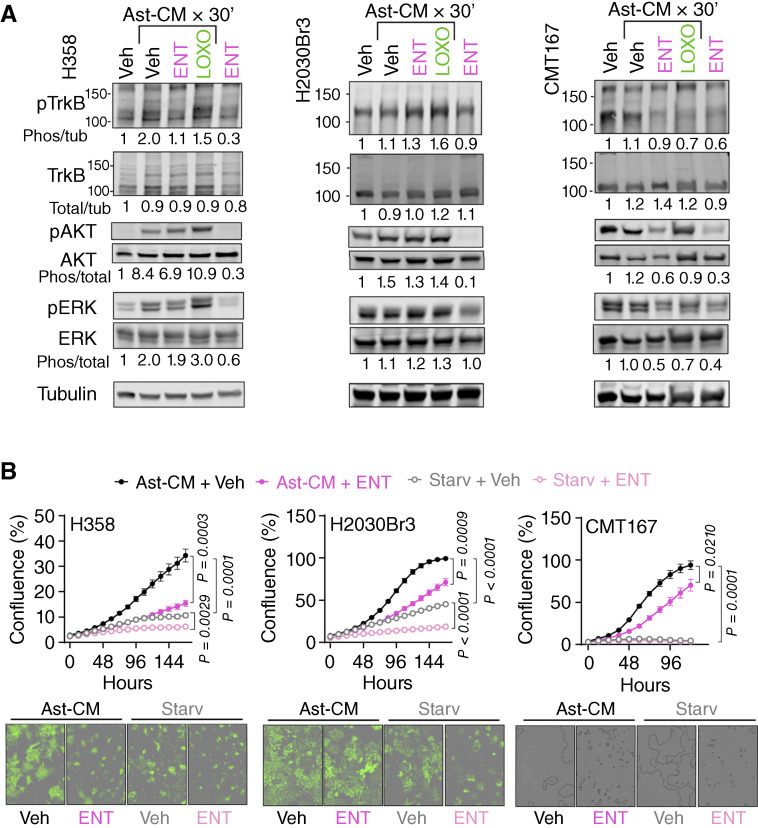
ENT decreases AKT and ERK activation and proliferation induced by astrocyte-conditioned media (Ast-CM) in NSCLC cells. **A,** Western blot of NSCLC cells serum-starved overnight, pretreated for 2 hours with 1 μmol/L ENT or LOXO101 (LOXO), then stimulated with Ast-CM (10×) for 30 minutes. Tubulin served as a loading control. Densitometric values were calculated as phospho-TrkB/tubulin, total TrkB/tubulin, phospho-AKT/total AKT, and phospho-ERK/total ERK ratios and were expressed relative to the vehicle (Veh) control group. **B,** Proliferation expressed as percent confluence (mean ± SEM) in NSCLC cells cultured in serum-starved media and treated with Ast-CM (10×) plus Veh (DMSO) or ENT (1 μmol/L). Data were analyzed by repeated-measures two-way ANOVA followed by Fisher Least Significant Difference (LSD) test (*n* = 5 wells per treatment).

We have reported that conditioned media from reactive astrocytes (Ast-CM) is a key source of growth factors, including, but not limited to, BDNF, and that it can, in a paracrine manner, activate prosurvival pathways and increase the proliferation of brain metastatic breast cancer cells (7, 33). Consistently, *NTRK2* downregulation blocked the proliferative effect of Ast-CM (Supplementary Fig. S3A), suggesting that the increased proliferation of H2030Br cells in response to astrocytes is, in part, dependent on *NTRK2*. To test how secreted factors from astrocytes affected signaling and proliferation, lung cancer cells were serum-starved overnight, pretreated with 1 μmol/L TKI, and then treated with Ast-CM (10×) or control (1% cs-FBS media). Confirming prior findings in BC cells, Ast-CM strongly activated prosurvival AKT signaling pathways in H358, LU65, and PC9Br3 cells, moderately in H2030Br3, and did not affect AKT activation in CMT167 cells, which showed strong levels of constitutive AKT activation (Fig. 3A; Supplementary Fig. S3B). As pretreatment with 1 μmol/L entrectinib, but not 1 μmol/L LOXO-101, modestly reduced signaling induced by Ast-CM across multiple cell lines despite a significant decrease when cells were treated with BDNF alone (Fig. 2A), we focused our next studies on defining the extent to which entrectinib could block or decrease activation of survival pathways in lung cancer cells in settings that mimic more complex interactions with the brain microenvironment. In H358 cells, entrectinib reduced Ast-CM–induced ERK activation but not AKT activation, whereas in H2030Br3 cells, entrectinib reduced endogenous but not Ast-CM–induced AKT or ERK activation. By contrast, entrectinib effectively blocked both AKT and ERK activation in CMT167 and PC9Br3 cells, whether alone or stimulated with Ast-CM (Fig. 3A; Supplementary Fig. S3B). Despite the differential effects of entrectinib on AKT and ERK activation among cell lines at the tested time points, sustained treatment with 1 μmol/L entrectinib significantly blocked cancer cell proliferation of vehicle-treated H358, H2030Br, and Lu65 cells and reduced their growth in response to Ast-CM to levels similar to those of vehicle-treated controls (Fig. 3B; Supplementary Fig. S3C). CMT167 and PC9Br3 cells also showed decreased proliferation in response to entrectinib when cultured with Ast-CM, yet this growth was still significantly higher than that of vehicle-treated cells, suggesting Ast-CM promotes additional growth in these cells through pathways that are not dependent on *NTRKs*.

**Figure 4.**
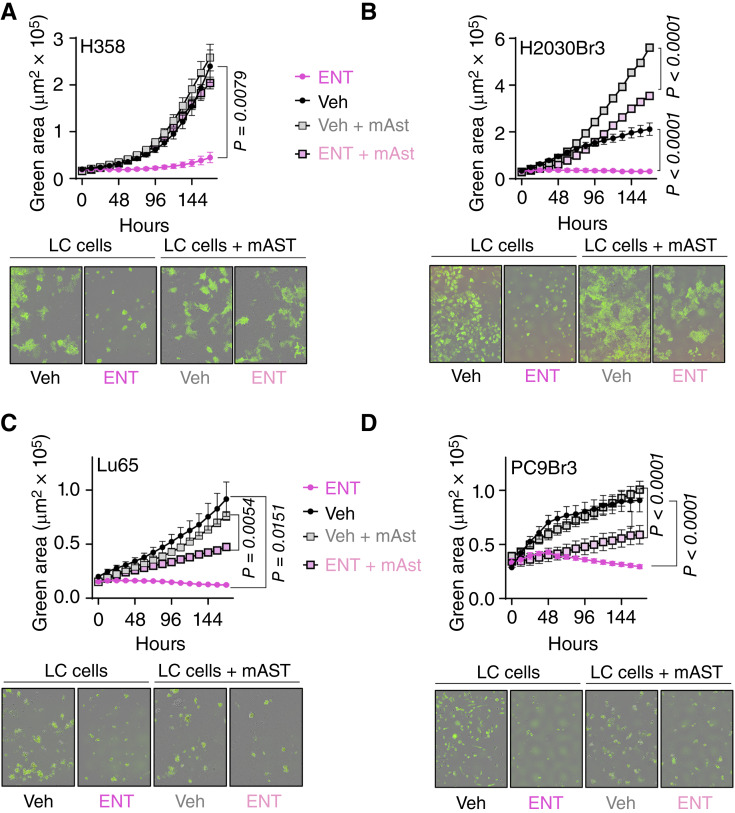
ENT reduces NSCLC cell proliferation in coculture with astrocytes. Proliferation was quantified as green area (μm^2^) using live-cell imaging. **A,** H358; (**B**) H2030Br3; (**C**) Lu65; and (**D**) PC9Br3 cells were cultured either in starvation media (2% cs-FBS) alone or over a 100% confluent monolayer of primary mouse astrocytes, in combination with vehicle (Veh; DMSO) or ENT (1 μmol/L). Data were analyzed by repeated-measures two-way ANOVA followed by Fisher LSD test (*n* = 5 wells per treatment). *P* values shown are the adjusted *P* values for Veh vs. Veh + Ent or Veh-mAst vs. ENT-mAst at the last time point.

Prior studies have shown that cell–cell contact with astrocytes can elicit prosurvival mechanisms in cancer cells different from those of secreted factors and can protect cancer cells from cytotoxic drugs (34, 35). To evaluate if entrectinib could decrease proliferation when cancer cells were in close contact with astrocytes, GFP^+^ cancer cells were plated on top of a monolayer of primary mouse astrocytes in media supplemented with 2% cs-FBS, and the green area was measured over time by live imaging. Surprisingly, only H2039Br3 cells showed a significant increase in proliferation when cocultured with astrocytes, whereas H358, PC9Br3, and Lu65 showed a similar ability to grow alone or in contact with astrocytes (Fig. 4A–D). Nonetheless, entrectinib significantly decreased the proliferation of H2030Br, Lu65, and PC9Br3 cancer cells, whether alone or cocultured with astrocytes (Fig. 4B–D). By contrast, H358 cells, which were sensitive to entrectinib when stimulated with Ast-CM (Fig. 3B), were rendered entrectinib-insensitive when cocultured with astrocytes, suggesting that cell contact with astrocytes may elicit additional survival pathways in these cells that are not targeted by entrectinib (Fig. 4A).

### ENT blocks brain metastatic colonization (prevention) and progression (therapeutic) of H203rM3 cells in preclinical models *in vivo*

**Figure 5.**
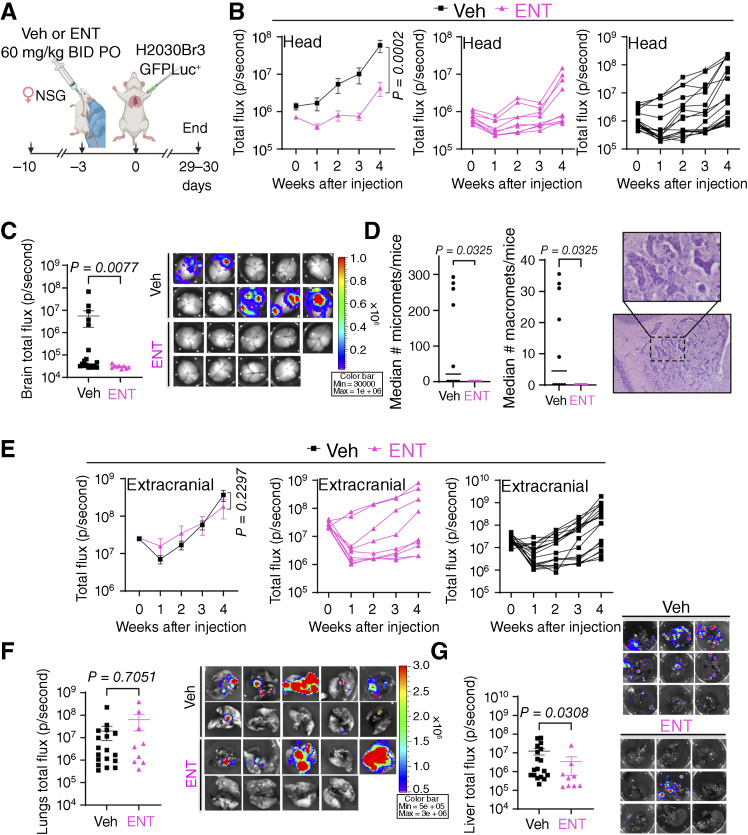
ENT prevents brain metastatic colonization of H2030Br3 cells. **A,** Experimental design: NSG female mice were randomized to receive vehicle (Veh) or ENT (60 mg/kg, twice daily (BID), orally (PO)]. Treatment began 3 days prior to the intracardiac injection of 200,000 H2030Br3 GFPLuc cells. Metastatic progression was monitored weekly by IVIS imaging, and mice were euthanized 4 weeks after inoculation. **B,** (Left) Mean ± SEM of total head flux by treatment; (middle and right) individual head flux values for ENT- and Veh-treated mice, respectively. **C,** Total *ex vivo* flux in the brain with representative IVIS images. **D,** Each dot represents the median number of micrometastases (<300 μm) and macrometastases (>300 μm) per mouse, Mann–Whitney test; representative hematoxylin and eosin images of macro and micro metastases forming a cluster are shown. **E,** (Left) Mean ± SEM of total extracranial flux by treatment; (middle and right) individual extracranial flux values for ENT-treated and Veh-treated mice. Group sizes are as follows: Veh = 18 and ENT = 9. **F,** Total *ex vivo* flux in lungs with representative IVIS images. **G,** Total *ex vivo* flux in livers with representative IVIS images. Statistical analysis: *In vivo* IVIS data were analyzed using two-way repeated-measures ANOVA; *P* values show adjusted *P* values for the last time point. *Ex vivo* data were analyzed using the Kruskal–Wallis test followed by Fisher LSD *post hoc* test.

We next sought to determine whether entrectinib could effectively block brain metastatic colonization (prevention of metastases) or decrease the progression of existing BM using an experimental model of intracardiac injection. This model bypasses cell dissemination from the PT but allows us to measure the ability of cancer cells to seed and colonize distant sites, including the brain. To determine if entrectinib could block seeding and early metastatic colonization, female NSG mice were pretreated with vehicle or 60 mg/kg twice a day entrectinib starting 3 days prior to intracardiac injection of 200,000 brain tropic H2030Br GFP-luc cells (which robustly form BM), and whole-body IVIS was used to measure metastatic burden over time (Fig. 5A). ENT significantly decreased head metastatic progression compared with vehicle-treated mice (Fig. 5B), which reflected a significant prevention of brain metastatic colonization as demonstrated by *ex vivo* IVIS as well as histologic quantification of metastases (Fig. 5C and D). Notably, entrectinib had minimal impact on cancer cell colonization at extracranial sites, with no significant differences in tumor burden in the lungs and a moderate decrease in metastatic burden in the liver, which can also produce BDNF (Fig. 5E–G; ref. 36), further supporting the notion that paracrine activation of *NTRK*, particularly TrkB, is critical to metastatic seeding and colonization in BDNF-expressing organs.

**Figure 6.**
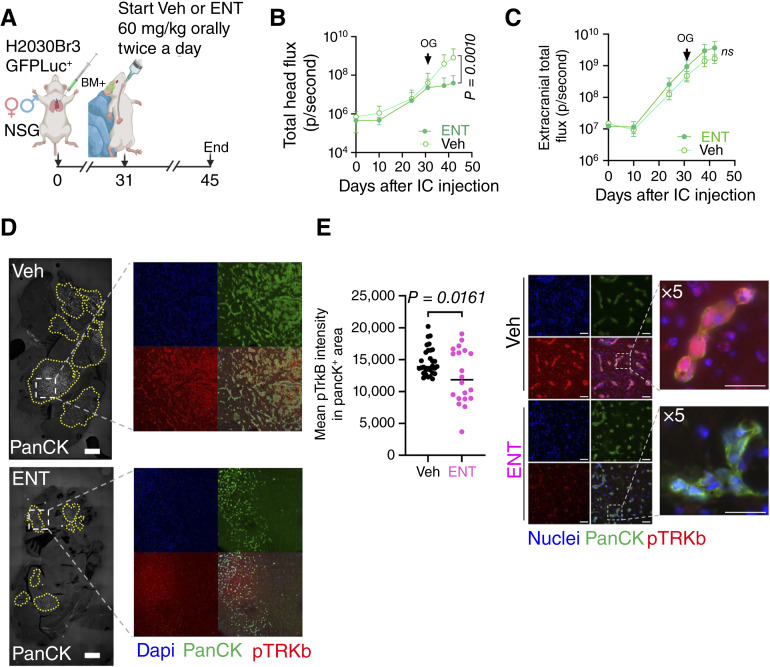
ENT reduces brain metastatic burden in a late-stage model of BMs. **A,** Experimental design: NSG female and male mice were injected intracardially with 200,000 H2030BrM3 GFPLuc cells. Head IVIS signal was monitored every 10 days. Mice showing a head signal at least twice the initial level were randomized to receive vehicle (Veh) or ENT (60 mg/kg, twice daily, orally). Group sizes are as follows: female (ENT) = 6, male (ENT) = 6, female (Veh) = 5, and male (Veh) = 7. Treatment lasted 14 days, and metastatic burden was monitored weekly. **B,** Total head flux for both sexes combined. **C,** Total extracranial flux for both sexes combined. *In vivo* IVIS data were analyzed using two-way repeated-measures ANOVA followed by Fisher LSD test for multiple comparisons. *P* values shown are adjusted *P* values for the last time point. **D,** Representative images of full brain sagittal sections from Veh and ENT-treated mice. Dotted lines mark the pan-cytokeratin positive (panCK^+^, gray tumor areas. Boxes show 5× magnification of p-TrkB (red), panCK (green), DAPI (nuclei), and merged images. Scale bars, 1,000 μm. **E,** Quantification of p-TrkB in 10 images/mice from 2 individual mice per group, at 40×. PanCK^+^ mask was generated to define tumor areas and converted into a region of interest; pTrkB mean fluorescence intensity was measured within panCK^+^ areas only. Representative images of fields were used for quantification. Scale bars, 50 μm.

We next tested the effectiveness of entrectinib to decrease brain metastatic progression in the setting of well-established BM. For this, H2030Br cells were injected into male and female NSG mice; BM were allowed to grow for 25 days, and mice with similar head tumor burden were randomized to either vehicle or entrectinib treatments (Fig. 6A). ENT decreased the progression of BM, as demonstrated by decreased head IVIS signal (Fig. 6B). Similarly to the preventive setting, entrectinib did not show a significant effect on extracranial metastatic progression, further suggesting that paracrine *NTRK* activation is critical to brain metastatic progression (Fig. 6C). Histologic analysis further shows that brains from vehicle-treated mice have large and multiple pan-cytokeratin–positive BM with high expression of p-TrkB, whereas entrectinib-treated mice contained smaller metastases with heterogeneous but significantly lower intensity of p-TrkB (Fig. 6D and E; Supplementary Fig. S4), confirming target inhibition *in vivo*.

## Discussion

Although the tumor-promoting role of *NTRK* receptors in lung cancer progression is well recognized, our studies further demonstrate a unique role for the brain activity of *NTRK* in lung cancer and the potential use of FDA-approved *NTRK* inhibitors in the prevention of CNS metastases for non-*NTRK* rearranged tumors. The fact that *NTRK* expression can be detected in PTs and does not significantly change in their BM suggests that testing for WT *NTRK* expression could serve as a criterion to define patients who could benefit from preventive strategies with *NTRK* inhibitors.

Mechanistically, we demonstrate that entrectinib can block the growth of lung cancer cells in the presence of astrocytes, cells that provide multiple survival signals and are important components of the brain niche (37, 38). This agrees with prior findings in preclinical models of breast cancer BM showing that astrocytes activate TrkB and also other receptors in cancer cells (i.e., EGFR), promoting proliferation, invasion, and brain colonization in breast cancer (7, 33). The observation that entrectinib is highly effective in blocking prosurvival signaling and cancer cell proliferation in multiple cell lines, when cells are cultured alone, in comparison with cells in coculture with astrocytes, highlights the important contribution from cells in the tumor microenvironment to the activation of growth and survival signals and the need to consider how tumor-host interactions alter therapeutic responses in BM. The significant effect of entrectinib blocking brain (the organ with the highest levels of BDNF) and, to a lesser extent, liver metastases (which express low levels of BDNF) without altering lung metastases highlights the potential to exploit the paracrine activation of TKIs in an organ-specific manner. Specifically, these data suggest *NTRK* inhibitors may have a role in protecting the brain (and potentially the liver) from metastatic spread, which could and should be explored clinically.

Although our data suggest a role for microenvironment activation of *NTRK* to support growth, it is likely that additional mechanisms contribute to the prometastatic function of *NTRK* in BM. For example, recent research indicates that primary brain tumors exploit BDNF-TrkB signaling to modulate cancer–neuron synapses, and inhibiting this pathway significantly suppresses tumor growth (39, 40). Given that neuron-to-lung and neuron-to-breast cancer synapses are essential for brain metastatic progression (41, 42), it is plausible that BDNF/TrkB-mediated synaptic plasticity and connectivity also play a role in the initiation of CNS metastases. In fact, the effective abolishment of BM when entrectinib is used prior to tumor injection suggests these early cancer–neuron or cancer–astrocyte interactions may be key for *NTRK* promotion of brain colonization. Nonetheless, our findings that entrectinib also delayed the progression of late-stage metastases suggest *NTRK* activation remains active throughout metastatic progression and can be targeted in a preventive and therapeutic setting.

Our results showed that entrectinib was more effective than the most selective TrkB inhibitors (such as cyclotraxin and ANA-12) in inhibiting the proliferation of lung cancer cells. It is likely that entrectinib, an ATP-competitive tyrosine kinase inhibitor with favorable intracellular bioavailability, is able to achieve sustained inhibition of the Trk kinase domain, whereas ANA-12 [which interferes with ligand binding (43)] and cyclotraxin-B [which acts as a negative allosteric modulator (44)] are non-ATP–competitive and may produce only partial or transient suppression of TrkB signaling. Moreover, although we focused on TrkB function here due to its increased expression among lung cancers and cell lines, it is possible that TrkA also plays a role in sensitivity to pan-Trk inhibitors. Elevated TrkA protein expression and increased NGF levels have been observed in squamous cell carcinoma compared with benign lesions and other malignant lung cancer histologic subtypes (45). It is therefore possible that even low-abundance TrkA, when stimulated, could contribute to residual AKT/ERK signaling when TrkB is selectively inhibited. Furthermore, recent studies indicate that suppression of wild-type *NTRK1* in murine lung cancer models enhances sensitivity to immunotherapy by promoting complement C3-mediated activation of T cells (46). As our studies were limited to human xenograft studies in immunecompromised mice, the extent to which entrectinib affects brain metastatic progression and its impact on antitumoral responses remain unknown.

Finally, among the tested *NTRK* inhibitors in clinical development, entrectinib blocked *NTRK* signaling at a lower effective dose, consistent with prior reports of increased IC_50_ for this compound. However, it is possible that higher effective doses of other TKIs have a similar ability to entrectinib to target brain metastatic colonization. Additional studies are needed to define whether LOXO-101 or new generation *NTRKs*, at their effective doses *in vivo*, have a similar ability to prevent seeding and colonization and decrease the progression of existing BM. Although the side effects of targeting the normal *NTRK* function in the brain, including pain sensation, appetite regulation, proprioception, memory, and learning, need to be considered, the potential value of *NTRK* inhibition for the prevention of BM may open new opportunities for the prevention of BM in patients at high risk.

## Supplementary Material

Supplementary Table 1 List of antibodies, sources, and working dilutions used in this study

Supplementary Figure 1 Downregulation of TrkB impairs BDNF-TrkB signaling and decreases proliferation induced by astrocytic conditioned media

Supplementary Figure 2 ENT decreased proliferation independently of AKT or ERK signaling pathway in some NSCLC.

Supplementary Figure 3 Effects of genetic or pharmacological TrkB inhibition on astrocyte-conditioned media-induced proliferation and signaling in NSCLC cells

Supplementary Figure 4 Workflow for quantifying pTrkB fluorescence intensity within GFP-defined ROIs.

## Data Availability

All data and protocols described in the article will be made available upon reasonable request within 6 years of publication.
