# Supplementary material for: Targeting Wild-type NTRK in NTRK Fusion–Negative Lung Cancer Decreases Brain Metastases
Source: Cancer Res Commun. 2026 Aug 4;6(8):1850–61. doi: 10.1158/2767-9764.CRC-25-0811 (PMC13436848; doi:10.1158/2767-9764.CRC-25-0811)
Supplement: Supplementary file 1 [file crc-25-0811_supplementary_figure_1_suppsf1.docx]

**Sup. Fig 1. Downregulation of TrkB impairs BDNF-TrkB signaling and decreases proliferation induced by astrocytic conditioned media**

**A.** Relative expression of NTRK2 in wild-type (WT) H2030Br3 cells (non-transduced), and H2030Br3 cells transduced with a non-targeting control shRNA (shNT) or shNTRK2. cDNA from human astrocytes was used as a positive control. **B.** quantification of total TrkB/ Vinculin of vehicle treated samples of sh-NT or sh-Trkb in 3 biological replicates. **C.** Representative western blot of TrkB downstream signaling in sh-NT or sh-TRKb H2030Br3 cells treated with vehicle (-) or BDNF (+) for 5 min. Densitometric values were calculated as phospho-TrkB/tubulin, total TrkB/tubulin, phospho-AKT/total AKT, and phospho-ERK/total ERK ratios, and were normalized to the vehicle control group
