# Supplementary material for: Targeting Wild-type NTRK in NTRK Fusion–Negative Lung Cancer Decreases Brain Metastases
Source: Cancer Res Commun. 2026 Aug 4;6(8):1850–61. doi: 10.1158/2767-9764.CRC-25-0811 (PMC13436848; doi:10.1158/2767-9764.CRC-25-0811)
Supplement: Supplementary file 3 [file crc-25-0811_supplementary_figure_3_suppsf3.docx]

**Sup. Fig. 3. Effects of genetic or pharmacological TrkB inhibition on astrocyte-conditioned media-induced proliferation and signaling in NSCLC cells**

**A.** Proliferation, expressed as fold change in percent confluence (mean ± SEM), in shNT and shNTRK2 H2030Br3 cells cultured under serum-starved conditions supplemented with 2.5% cs-FBS or treated with astrocyte-conditioned media (AST-CM, 10X). Data were analyzed by repeated-measures two-way ANOVA followed by Fisher’s LSD test (n = 5 wells per condition). **B**. TrkB signaling in NSCLC cells serum-starved overnight, pretreated for 2 hours with 1 μM Entrectinib (ENT) or LOXO101 (LOXO), followed by stimulation with Ast-CM (10X) for 30 minutes. Tubulin was used as a loading control. **C.** Proliferation expressed as percentage of confluence in NSCLC cells cultured in serum-starved media and treated or not with Ast-CM (10X) in combination with vehicle (DMSO) or ENT at 1 μM. Data were analyzed using repeated-measures two-way ANOVA followed by Fisher’s LSD test (n = 5 wells per treatment).
